# Responses of differential metabolites and pathways to high temperature in cucumber anther

**DOI:** 10.3389/fpls.2023.1131735

**Published:** 2023-04-14

**Authors:** Lin Chen, Zhaojun Liang, Shuyan Xie, Wenrui Liu, Min Wang, Jinqiang Yan, Songguang Yang, Biao Jiang, Qingwu Peng, Yu’e Lin

**Affiliations:** ^1^ Vegetable Research Institute, Guangdong Academy of Agricultural Sciences, Guangzhou, China; ^2^ Guangdong Key Laboratory for New Technology Research of Vegetables, Guangzhou, China

**Keywords:** cucumber, high temperature stress, pollen fertility, metabolomics, starch and sucrose metabolism

## Abstract

Cucumber is one of the most important vegetable crops, which is widely planted all over the world. Cucumber always suffers from high-temperature stress in South China in summer. In this study, liquid chromatography–mass spectrometry (LC-MS) analysis was used to study the differential metabolites of cucumber anther between high-temperature (HT) stress and normal condition (CK). After HT, the pollen fertility was significantly reduced, and abnormal anther structures were observed by the paraffin section. In addition, the metabolomics analysis results showed that a total of 125 differential metabolites were identified after HT, consisting of 99 significantly upregulated and 26 significantly downregulated metabolites. Among these differential metabolites, a total of 26 related metabolic pathways were found, and four pathways showed significant differences, namely, porphyrin and chlorophyll metabolism; plant hormone signal transduction; amino sugar and nucleotide sugar metabolism; and glycine, serine, and threonine metabolism. In addition, pollen fertility was decreased by altering the metabolites of plant hormone signal transduction and amino acid and sugar metabolism pathway under HT. These results provide a comprehensive understanding of the metabolic changes in cucumber anther under HT.

## Introduction

Temperature is an important factor in plant growth, and each plant has its own suitable temperature for growth. Plant growth and development and yield will be adversely affected if the temperature is higher than the suitable temperature. Heat damage has recently become more severe, particularly with the intensification of the global “greenhouse effect” (Rahman et al., 2018; Ahmad et al., 2021; Abbas, 2022). The crop growing season is often affected by heat damage in summer, which affects crop growth and yield (Cao et al., 2022). Crop plants make a range of responses to cope with heat stress. For example, the fluidity of the cell lipid membrane is significantly enhanced under high-temperature stress, which can cause electrolyte leakage, reactive oxygen species generation, and oxidative damage (Bokszczanin and Fragkostefanakis, 2013; Zhang et al., 2022a). To cope with heat damage, plants accumulate metabolites such as antioxidants, osmoprotectants, and heat shock proteins through different pathways and protective enzymes and antioxidants in the chloroplasts and mitochondria (Bohnert et al., 2006; Moore et al., 2021). Heat shock proteins play roles in protecting and repairing the damaged protein, as well as regulating cellular redox status (Wang et al., 2004; Zhong et al., 2013; Masoomi-Aladizgeh et al., 2021). Other major factors that respond to high-temperature stress include ion transporters, osmotic protective agents, free radical scavenging agents, and all kinds of stress response proteins, and they participate in the signal level and transcriptional control factor (Wang et al., 2004). Thus, to ensure continued food security or crop yield, a practical strategy is needed to analyze the mechanism of crop heat resistance, cultivate heat-resistant crops, and maintain high yield with high temperature.

Cucumber (*Cucumis sativus* L.) is one of the most important vegetables in the world. Cucumber often suffers from high-temperature stress in summer, which leads to premature leaf senescence, reduced photosynthesis and respiration, damaged cell membrane system, and decreased pollen vigor, affecting fruit quality and shape and yield (Sun et al., 2018). *CsCaM3* is a plant calmodulin gene that improves cucumber high-temperature tolerance by regulating a series of high-temperature-related genes (Yu et al., 2018). In addition, the exogenous glutathione was thought to reduce high-temperature stress damage. Exogenous glutathione can regulate osmolytes, photosynthesis, and antioxidant systems to improve the high-temperature tolerance of cucumber (Ding et al., 2016). Although high temperature affects both the vegetative and reproductive stages, the reproductive stage is more sensitive to high-temperature stress than the vegetative stage (Hedhly et al., 2009; Prasad et al., 2017; Lohani et al., 2020), and cucumber is no exception. Notably, male cucumber flowers were more sensitive to high temperature than female flowers (Miao and Li, 2001), and the pollen fertility and viability were observed to be significantly decreased (Miao, 2000).

Metabolomics is a comprehensive and systemic discipline developed after genomics, RNA-seq, and proteomics, which can be applied to study the endogenous metabolites of small molecules in living organisms (Li and Gaquerel, 2021), and it is correlated directly with downstream phenomics. Recently, metabolomics has been widely used in studies of plant growth and development (Du et al., 2020; Hu et al., 2021), biotic stress (Silva et al., 2021), and abiotic stress (Kumar et al., 2021). Many metabolism pathways were found to respond to high temperatures in *Paspalum wettsteinii*, including sugar metabolism, amino acid metabolism, and auxin metabolism (Zhao et al., 2022). Abiotic stress can induce the biosynthesis and accumulation of related osmotic fluid, including sugars, amino acids, and secondary metabolites. These metabolites can maintain the osmotic potential in cells, which is the main adaptation strategy of plants’ resistance to abiotic stress (Jia et al., 2019). Sugar metabolism is widely considered an energy source for plant development and plays an essential role in male reproductive development in higher plants. The disturbance of starch and sucrose metabolism will cause male sterility at the reproductive stage of plants (Liu et al., 2021b). The hormonal pathways are disordered after high-temperature stress and cause male sterility (Khan et al., 2020). The auxin transport in the anther is affected under high-temperature stress, and the content of auxin is disordered, which leads to a reduction in male fertility (Yao et al., 2018). Furthermore, metabolomics has also been widely used to study the different metabolites in cucumber (He et al., 2020; Liu et al., 2021a; Pan et al., 2022). Li et al. (2018) clarified that metabolites and metabolic pathways, related to mitigating drought stress by increasing the content of CO_2_, were different under a series of drought stress. However, until now, studies involving metabolomics analysis of male cucumber flowers under high-temperature stress have been limited.

It is well known that the reproductive development of cucumber is affected by high-temperature stress, which then leads to a reduced yield. Moreover, the summer temperature is high in South China, and thus, breeding heat-resistant cucumber varieties has become one of the most effective and economical ways to increase cucumber yield during summer in South China. However, to our best knowledge, metabolic research about male cucumber flowers under heat stress is still lacking until now. Therefore, the heat-resistant abilities of male cucumber flowers need to be further explored in the future, which is of great significance for the improvement of breeding heat-resistant cucumber cultivars. Based on our previous research on the dynamic changes of gene expression on male cucumber flower development under high-temperature stress (Chen et al., 2021b), this study will use metabolomics to further explore the changes of metabolites in the mature pollen stage of cucumber, providing a new idea for research under normal growth conditions and high-temperature stress. The results will provide us a better understanding of how male cucumber flowers, especially its mature pollen, make a reaction at the metabolic level under high-temperature stress, as well as provide a theoretical foundation for heat-resistant cucumber breeding to minimize the damage caused by high-temperature stress.

## Materials and methods

### Plant material and sampling

The cucumber seeds were immersed in water at room temperature for 4-5 h and allowed to sprout overnight under a wet and dark environment. Then, the germinated seeds were cultivated in plant pots and grown in a growth cabinet. When the cucumber plants formed the sixth true leaves, they were moved into a phytotron with 70% relative humidity under normal conditions (CK, 12 h at 28°C in the light/12 h at 25°C in the dark) or high-temperature (HT, 12 h at 38°C in the light/12 h at 30°C in the dark) stress (Chen et al., 2021b). These temperature treatments were stopped until we collected enough male flowers for observations of the anther structure and pollen fertility and for the metabolomics experiments. The mature anthers of three biological replicates were dissected from male flowers and harvested for metabolomics analysis.

### Cytological observation

The investigation of pollen fertility was performed as previously described (Chen et al., 2021b). The mature male flowers were obtained and quickly put in Carnoy’s solution (ethanol:acetic acid = 3:1) for over 24 h, and they were kept in 70% ethanol after washing three times. The anther was removed from the inflorescences and placed in 1% iodine potassium (I_2_-KI), and the normal and abnormal pollens were observed on a microscope (Motic BA200).

Anther structure survey by paraffin section was conducted according to the methods provided by Chen et al. (2021b). The anthers at mature stages were collected and kept in formaldehyde–acetic acid–ethanol (FAA) (70% ethanol:acetic acid:methanol = 89:6:5) solution for 48 h. Subsequently, the anthers were dehydrated by a series of ethanol concentrations according to the protocol of the manufacturers, and then the dehydrated anthers were embedded in paraffin. The embedded anthers were put in a microtome (Leica RM2235) and then cut with a cross-section thickness of 2 to 5 μm. Finally, the cross sections of the anthers were stained in 0.05% toluidine blue (m/v) and covered with a slide cover for observation. The finished cross sections were observed by a microscope (Motic BA200).

### Metabolomics experiments and data analysis

The six collected anthers were quickly transferred to liquid nitrogen and kept at −80°C for the extraction of metabolites by 80% methanol. The metabolites of three biological samples were extracted for liquid chromatography–mass spectrometry (LC-MS) analyses following manual instructions (Want et al., 2013). LC-MS/MS analyses were performed using an ExionLC™ AD system (Sciex) coupled with a QTRAP^®^ 6500+ mass spectrometer (Sciex) in Novogene Co., Ltd. (Beijing, China), according to standard metabolic operating procedures (Luo et al., 2015). The metabolites were determined based on the Novogene in-house database by using multiple reaction monitoring (MRM). These metabolites were annotated and based on public databases, including the Human Metabolome Database (HMDB) (http://www.hmdb.ca/), Kyoto Encyclopedia of Genes and Genomes (KEGG) (http://www.genome.jp/kegg/), MassBank (http://www.massbank.jp/), and LIPID MAPS (http://www.lipidmaps.org/). Partial least squares discriminant analysis (PLS-DA) and univariate analysis (*t*-test) were applied to calculate the values of variable importance in projection (VIP) and statistical significance (*P*-value), respectively. Significantly differentially expressed metabolites (VIP >1, *P*-value <0.05, and fold change ≥2 or fold change ≤0.5) were screened and subjected to further analysis.

## Results

### Phenotype of pollen on high-temperature stress

**Figure 1:**
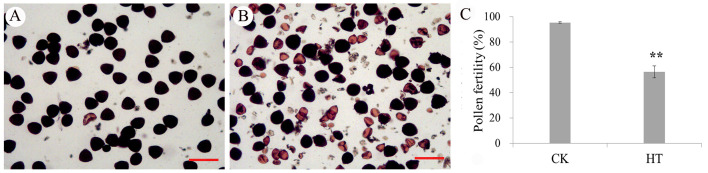
Pollen fertility of normal condition and high-temperature stress. **(A)** Pollen fertility of normal condition; **(B)** pollen fertility of high-temperature stress; **(C)** pollen fertility. Bar = 100 μm.

**Figure 2:**
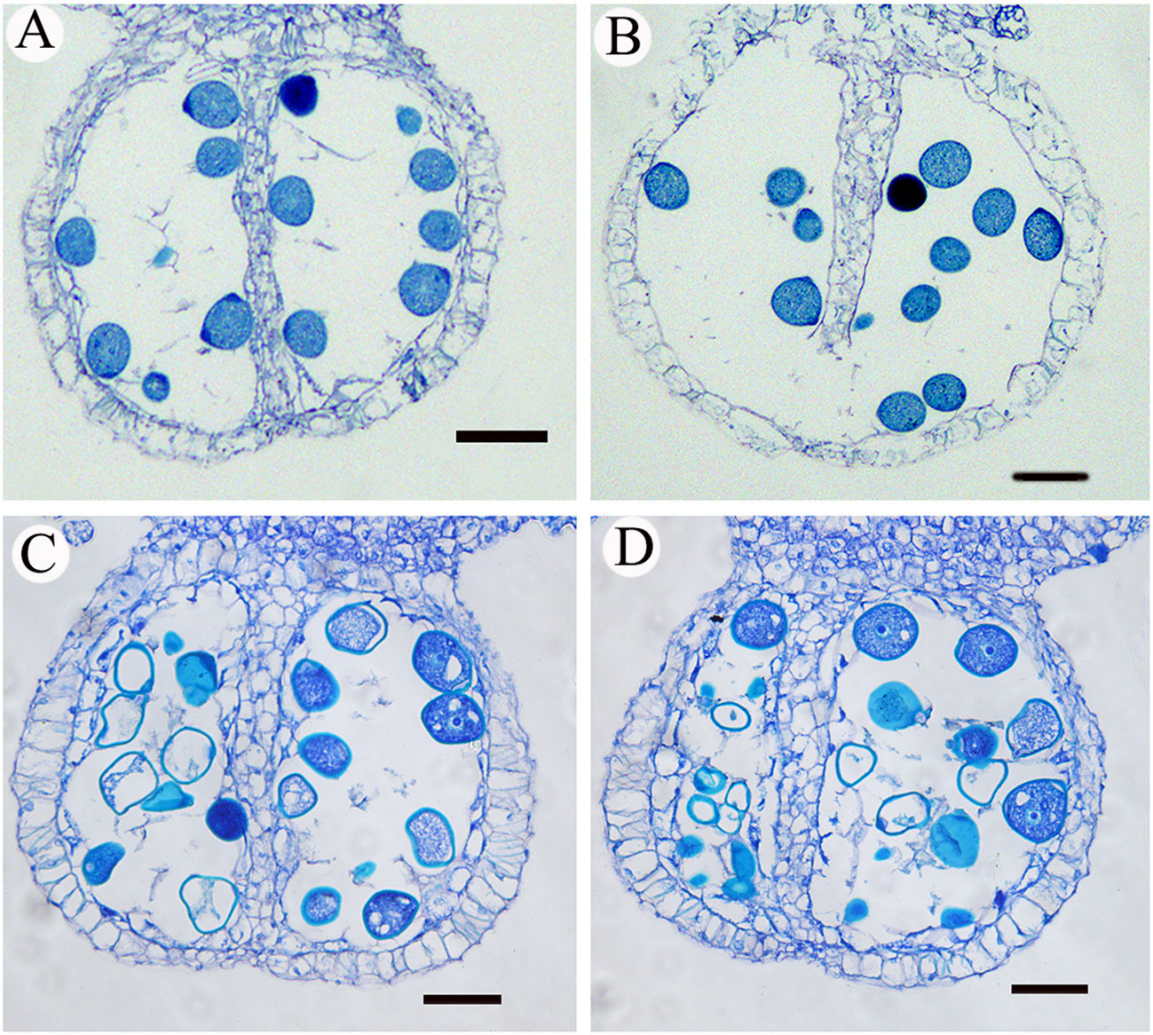
The cross-section analysis of mature anther. **(A, B)** Anther cross sections of the mature stage in normal condition; **(C, D)** the cross-sections of mature anther with high-temperature stress. Bar = 50 μm.

In this study, pollen fertility was detected between CK and HT. A large amount of abortive pollen grains appeared at the mature stage under high-temperature stress by I_2_-IK staining, but there were almost no aborted pollen grains in the normal condition (**Figures 1A, B**). The pollen fertility of HT was 56.22% and obviously lower than that in CK (95.25%) (**Figure 1C**). Next, the mature anther structures were observed by the paraffin section between CK and HT. The section results showed that the anther tapetum of CK was normally degraded and the pollen grains were normally developed (**Figures 2A, B**), whereas a large number of insufficient pollen grains and delayed degradation tapetum were found in HT (**Figures 2C, D**). These cytological results showed that the development of mature anther was significantly impaired in HT stress compared with that in CK.

### Overview of metabolomics profiling

To study the possible molecular differences of cucumber pollen at the mature stage under high-temperature stress, we used metabolomics analysis to investigate the differential metabolites. A total of 1,224 metabolites were obtained, which were classified into 57 classes (**Table S1**). The top 10 metabolites were classified into amino acids and derivatives, flavonoids, carbohydrates and derivatives, nucleotides and derivatives, organic acids and derivatives, terpenoids, fatty acyls, flavones and flavonols, cinnamic acids and derivatives, and phospholipids. The number of amino acids and derivatives is the most abundant and reached 200. The number in the abovementioned classes was 118, 79, 73, 71, 51, 48, 37, 36, and 36, respectively (**Table 1**). In addition, principal component analysis (PCA) of all the samples and quality control (QC) samples was executed. As shown in **Figure S1A**, the distribution of the QC sample points was relatively clustered, indicating that the QC sample differences were relatively small. On the other hand, it also indicated that the sampling method for metabolomics analysis was stable and the resulting data quality is relatively high in this study. The cumulative contribution rate reaches 61.85% (PC1: 45.73%, PC2: 16.12%), and the distribution of samples in different treatments was obviously separated (**Figure S1B**). These results indicated that metabolites of anther under HT were significantly distinguished from those under CK.

**Table 1:** The type and quantity of identified metabolites between CK and HT.

Class	Number	Class	Number
Amino acids and derivatives	200	Quinones	7
Flavonoids	118	Pentose phosphates	7
Carbohydrates and derivatives	79	Glycosides	7
Nucleotides and derivatives	73	Ketones	7
Organic acids and derivatives	71	Phenolamides	6
Terpenoids	51	Glycerolipids	6
Fatty acyls	48	Lignans	6
Flavones and flavonols	43	TCA cycle	6
Cinnamic acids and derivatives	37	Sugar alcohols	6
Phospholipid	36	Sugar acids and derivatives	6
Alkaloids and derivatives	36	Terpene	6
Amines	30	Chalcones and dihydrochalcones	5
Phenols and derivatives	25	Quinolines and derivatives	5
Organoheterocyclic compounds	24	Steroids and steroid derivatives	5
Benzene and substituted derivatives	22	Lactones	5
Phytohormones	22	Polyphenol	4
Flavanones	20	Pyrimidines and pyrimidine derivatives	3
Vitamins	20	Cholines	2
Anthocyanins	19	Catechin derivatives	2
Coumarins and derivatives	19	Ethers	2
Indoles and derivatives	18	Polyamine	1
Benzoic acids and derivatives	15	Phenolic acids	1
Phenylpropanoids and polyketides	14	Glycerophospholipids	1
Phenylpropanoids	14	Carotenoids	1
Purines and purine derivatives	13	Prenol lipids	1
Isoflavonoids	13	Isoquinolines and derivatives	1
Alcohols and polyols	12	Organosulfur compounds	1
Carbonyl compounds	11	Saponin	1
Pyridines and derivatives	10		

### Differential metabolites under high-temperature stress

**Figure 3:**
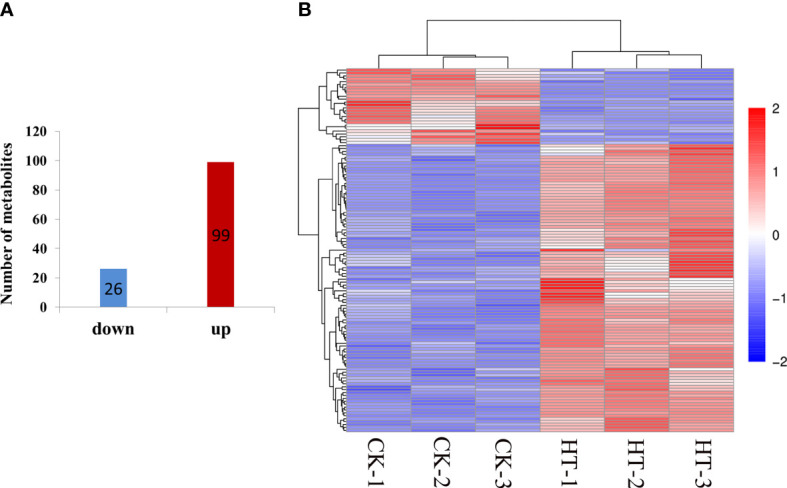
Differential metabolites in high-temperature stress compared with the normal condition at mature anther stages. **(A)** Number of up- and downregulated metabolites in high-temperature stress compared with normal condition. **(B)** Expression patterns of differential metabolites in high-temperature stress compared with normal condition.

**Figure 4:**
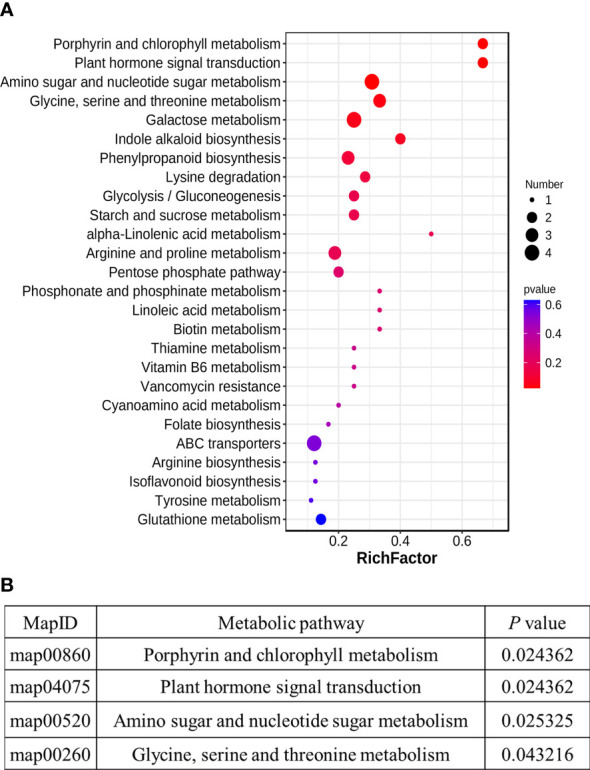
The Kyoto Encyclopedia of Genes and Genomes (KEGG) analyses of differential metabolites between normal condition and high-temperature stress. **(A)** KEGG enrichment of annotated metabolites from normal condition and high-temperature stress. The *y*-axis indicates the KEGG pathway and the *x*-axis indicates the enrichment factor. **(B)** Significant difference in KEGG pathway enrichment.

**Figure 5:**
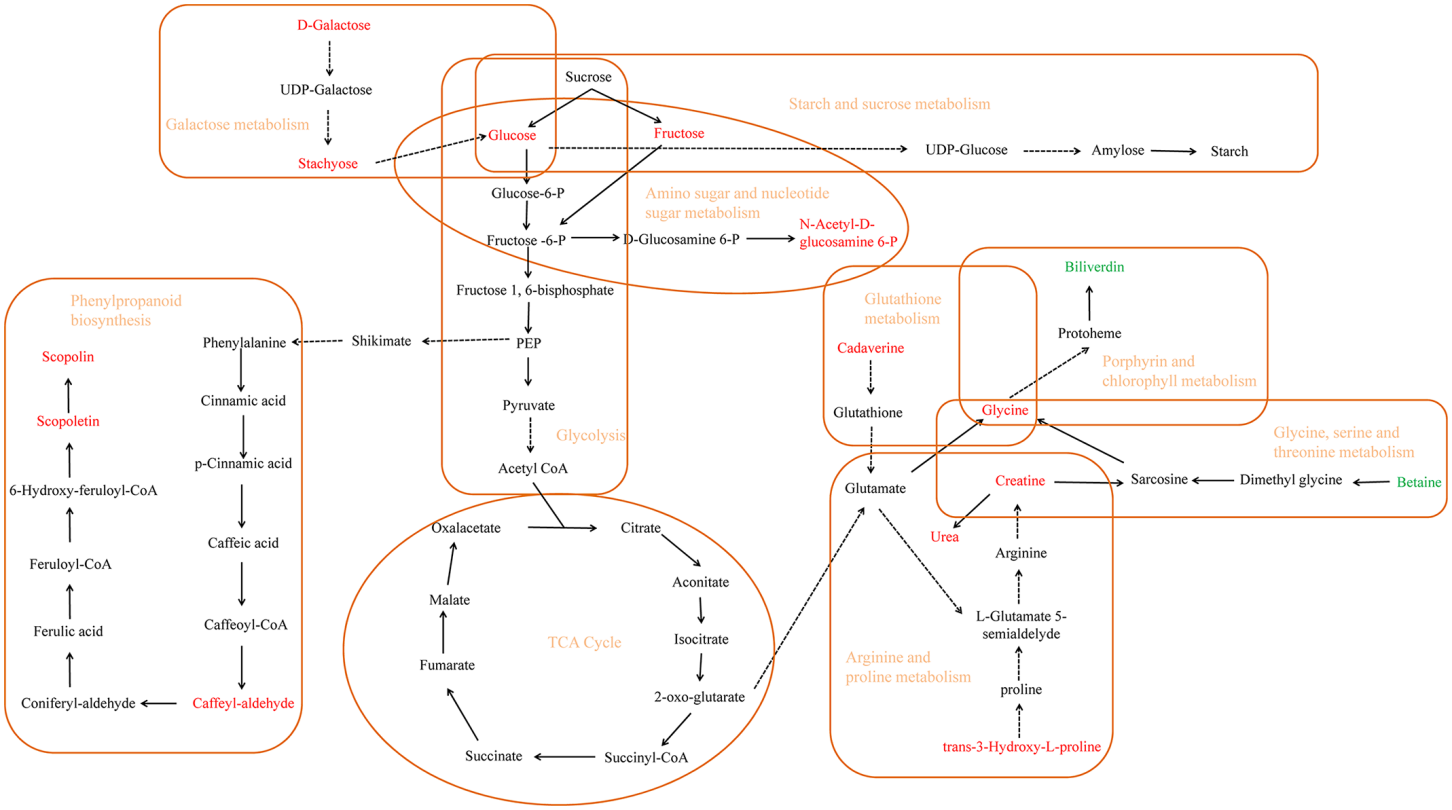
The main KEGG pathway responses to high-temperature stress. Red indicates upregulation, green indicates downregulation, and black indicates no difference. Solid and dashed lines indicate single- and multistep reactions, respectively.

We performed the PLS-DA model to study which metabolites have significant differences under HT. The differential metabolites were obviously classed between HT and CK (**Figure S2A**), which was consistent with the PCA analysis results. The *R* ^2^ regression line is located above the *Q* ^2^ regression line, and the intercept is −1.66 (less than 0) between the *y*-axis and the *Q* ^2^ regression line (**Figure S2B**), indicating that this model can describe the samples well. The differential metabolites were identified based on three filter conditions: VIP ≥1.0, *P*_value *<*0.05 in the *t*-test, and fold change ≥2 or fold change ≤0.5. A total of 125 differential metabolites were identified (**Table S2**), consisting of 99 upregulated and 26 downregulated metabolites (**Figure 3**). These metabolites were classified into 30 groups, and amino acids and derivatives (22) and carbohydrates and derivatives (15) were the two top groups (**Table 2**). Interestingly, only one metabolite was downregulated in these two groups, respectively. In addition, the results of the KEGG analysis demonstrated that 125 differential metabolites were enriched in 26 metabolic pathways, and amino sugar and nucleotide sugar metabolism; galactose metabolism; ABC transporters; glycine, serine, and threonine metabolism; phenylpropanoid biosynthesis; arginine and proline metabolism; and starch and sucrose metabolism were the top 7 pathways (**Figure 4A**, **Table S3**). These differential metabolites and metabolic pathways may provide crucial information on how cucumber anther responds to HT stress. Moreover, four metabolic pathways with significant differences were detected, namely, porphyrin and chlorophyll metabolism; plant hormone signal transduction; amino sugar and nucleotide sugar metabolism; and glycine, serine, and threonine metabolism (**Figure 4B**, **Table S3**). In addition, we constructed a comprehensive systemic metabolic pathway diagram under HT stress according to KEGG enrichment data (**Figure 5**).

**Table 2:** The type and quantity of identified differential metabolites between CK and HT.

Class	Upregulated	Downregulated
Amino acids and derivatives	21	1
Amines	2	NA
Phenylpropanoids and polyketides	2	NA
Phenylpropanoids	3	1
Benzene and substituted derivatives	2	1
Benzoic acids and derivatives	6	2
Pyridines and derivatives	2	NA
Polyphenol	1	1
Phenolamides	1	NA
Phenols and derivatives	3	2
Phenolic acids	NA	1
Glycerolipids	2	NA
Nucleotides and derivatives	1	NA
Anthocyanins	2	NA
Flavones and flavonols	1	2
Quinones	NA	1
Flavonoids	8	1
Phospholipid	2	NA
Lactones	1	NA
Carbonyl compounds	NA	1
Cinnamic acids and derivatives	1	2
Alkaloids and derivatives	1	1
Glycosides	2	
Carbohydrates and derivatives	14	1
Terpenoids	1	NA
Ketones	NA	1
Vitamins	1	1
Prenol lipids	1	NA
Coumarins and derivatives	3	NA
Isoflavonoids	2	NA
Indoles and derivatives	1	2
Organic acids and derivatives	2	2
Organoheterocyclic compounds	2	1
Fatty acyls	6	NA
Phytohormones	2	1
Total	99	26

NA, not available.

**Figure 6:**
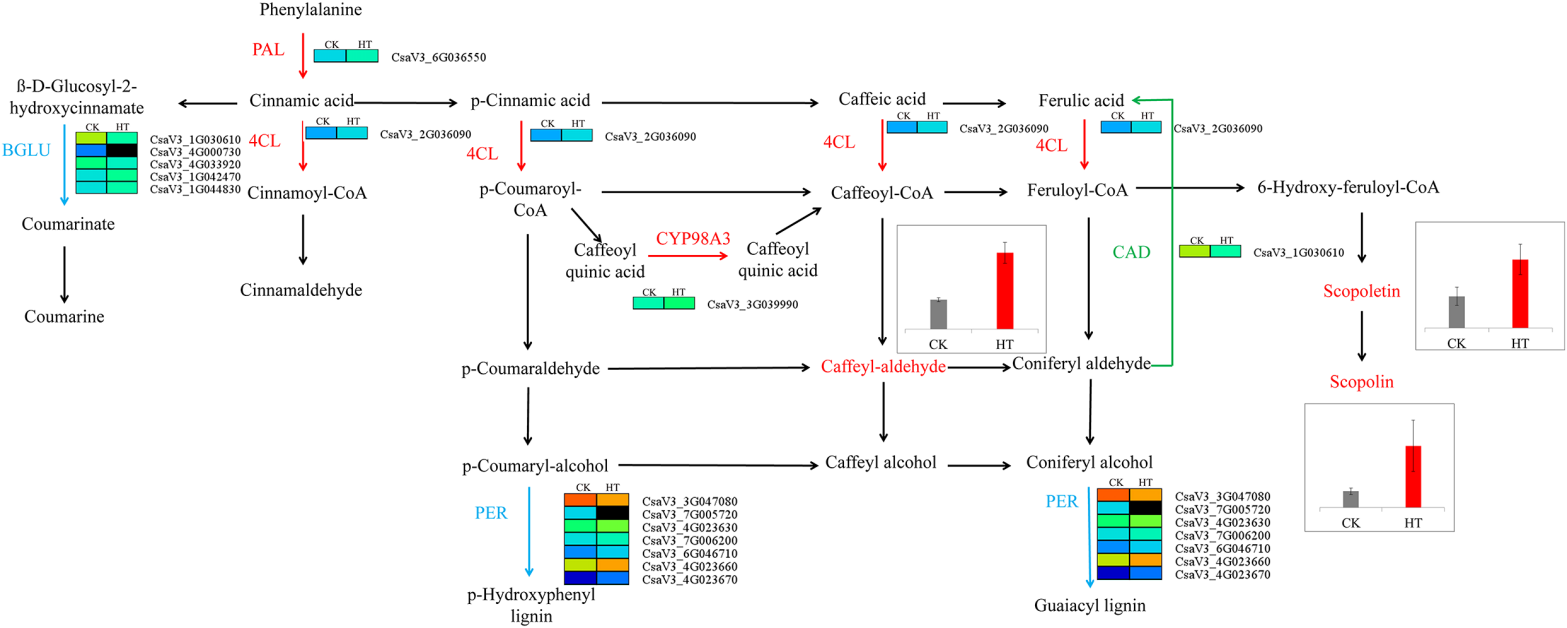
Predicted phenylpropanoid biosynthesis pathways under high-temperature stress. PAL, phenylalanine ammonia-lyase; 4CL, 4-coumarate-CoA ligase; BGLU, beta-glucosidase; PER, peroxidase.

**Figure 7:**
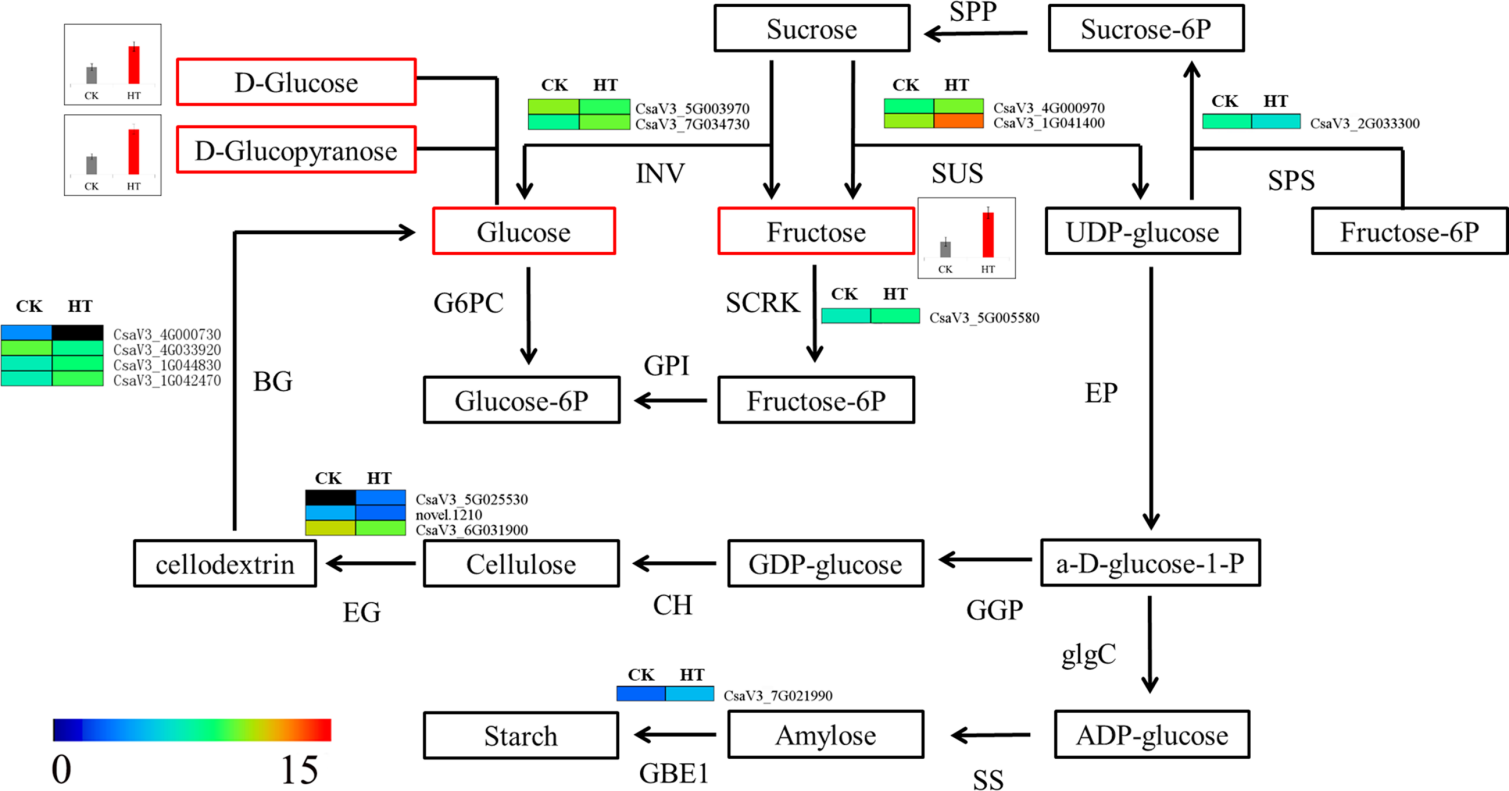
Predicted carbohydrate metabolism pathways under high-temperature stress. SPP, sucrose-6-phosphatase; INV, invertase; SUS, sucrose synthase; SPS, sucrose-phosphate synthase; G6PC, glucose-6-phosphatase; SCRK, fructokinase; GPI, glucose-6-phosphate isomerase; EP, ectonucleotide pyrophosphatase; glgC, glucose-1-phosphate adenylyltransferase; SS, starch synthase; GBE1, glucan branching enzyme; CH, cellulose synthase; EG, endoglucanase; BG, beta-glucosidase.

In our previous study, we acquired RNA-seq profiling of male flower development under HT stress, and 1,677 differentially expressed genes were identified at the mature pollen development stage (Chen et al., 2021b). Herein, a joint analysis of RNA-seq and metabolomics data was adopted to uncover the molecular mechanism of cucumber pollen responses to HT at the transcriptional and metabolic levels (**Table S4**). Among the top 7 KEGG pathways and significantly differential KEGG pathways, three KEGG pathways, namely, phenylpropanoid biosynthesis, plant hormone signal transduction, and starch and sucrose metabolism, had the largest number of differentially expressed genes (**Table S4**). In the phenylpropanoid biosynthesis pathways, scopoletin (Com_1169_pos), scopolin (Com_621_pos), and caffeic aldehyde (Com_684_pos) were upregulated in HT than in CK, and 15 differentially expressed genes were identified in our previous RNA-seq data (**Table S5**, **Figure 6**). The upregulated metabolites, including D-glucopyranose (Com_255_neg), D-glucose (Com_288_neg), and fructose (Com_472_neg), were involved in the pathway of starch and sucrose metabolism, and 14 differentially expressed genes were found in previous RNA-seq data, including nine upregulated and six downregulated differentially expressed genes (**Figure 7**, **Table S6**). In the plant hormone signal transduction pathway, indole-3-acetic acid (Com_670_pos) and jasmonic acid (Com_175_neg) were found upregulated in HT than in CK, and a large number of differentially expressed genes were found. In the auxin pathway, 16 differentially expressed genes were explored. The jasmonic acid pathway was composed of two differentially expressed genes: one upregulated and one downregulated gene (**Table S7**).

## Discussion

Cucumber is a kind of vegetable crop that prefers a warm climate but is not heat-tolerant, whose optimal growth temperature ranges from 25°C to 30°C. When the temperature exceeds 35°C, cucumber will encounter heat damage, and reproductive growth is more vulnerable than vegetative growth. Furthermore, the impairment of male cucumber flowers is more severe as compared with female flowers (Miao and Li, 2001). Specifically, high-temperature stress affects male flower development (Zhang et al., 2021; Zhang et al., 2022b), including damaged anther structure, decreased pollen fertility, reduced pollen vitality, and disordered reproductive process (Shi et al., 2013; Kumar et al., 2019). In cucumber, pollen fertility is lessened by high temperature (Miao, 2000; Chen et al., 2021b). In the current study, high temperature led to the production of a lot of sterile pollen, caused a large number of insufficient pollen grains, and delayed tapetum degradation. These results were consistent with previous a study, which suggested that high-temperature stress will affect anther development and reduce pollen fertility (Chen et al., 2021b).

The amino acids take part in responses to abiotic stresses in higher plants (Du et al., 2020). Amino acids and derivatives have important roles in keeping the structural integrity of intracellular proteins and osmoregulation. A number of amino acids (threonine, isoleucine, leucine, asparagine, malate, valine, alanine, etc.) were accumulated in *Arabidopsis* and rice by heat stress (Kaplan et al., 2004; Yamakawa and Hakata, 2010). Here, differential metabolites consisting of a total of 22 amino acids and derivatives were found between high-temperature stress and normal condition, which composed the largest classes (**Table 2**). These metabolites were mainly involved in glycine, serine, and threonine metabolism and arginine and proline metabolism. Proline, which is a compatible solute, is essential for adjusting osmotic pressure and protects the cell structure under abiotic stress (Slama et al., 2015). The levels of glycine and trans-3-hydroxy-L-proline significantly increased during high-temperature stress in cucumber anther. Sugars have also been considered to play an important role in osmotic adjustment, and they accumulated for resistance to abiotic stresses (Ma et al., 2009). In this study, a total of 15 carbohydrates and derivatives were found between high-temperature stress and normal condition. Of these carbohydrates and derivatives, 14 metabolites were upregulated, and only Com_971_pos (tuberonic acid hexoside) was downregulated in HT (**Table 2**, **Table S2**). These results suggest that cucumber anther maintained osmotic balance by increasing the levels of amino acids and derivatives as well as carbohydrates and derivatives. Sugar metabolism is considered energy for plant growth and development and is essential for male reproductive development in higher plants. Any disturbances in starch and sucrose metabolism will cause male sterility at the reproductive stage of plants (Goetz et al., 2001; Liu et al., 2021b). The starch in the anther decreased under high-temperature stress, and it led to a reduction of pollen germination and to male sterility (Begcy et al., 2019). In addition, the accumulation of glucose and fructose in the anther is one main reason for the low pollen fertility in autotetraploid rice (Chen et al., 2018). Herein, three products from the starch and sucrose metabolism pathway, two D-glucose (Com_255_neg and Com_288_neg) and a fructose (Com_472_neg), were significantly upregulated in high-temperature stress. In our previous study (Chen et al., 2021b), 14 differentially expressed genes were found at the mature stage in high-temperature stress than in normal condition (**Figure 6**, **Table S5**). These abrupt expressions of genes and the upregulation of metabolites of two D-glucose and a fructose lead to failure of starch synthesis under high-temperature stress and may cause pollen sterility. Our results were consistent with the increasing accumulation of glucose and fructose after high-temperature stress (Begcy et al., 2019).

The phenylpropanoid biosynthesis pathway is activated under abiotic stress (high temperature, cold, drought, and salinity) and leads to the accumulation of phenolic compounds, which have the potential to remove harmful reactive oxygen species (ROS) (Sharma et al., 2019). It was reported that caffeic acid and ferulic acid of the phenylpropanoid biosynthesis pathway play an important role in response to high-temperature stress in turfgrass species (Wang et al., 2019). Sun et al. (2022) found that phenylpropanoid metabolism (flavonoid metabolism and lignin synthesis) plays a key role in regulating male fertility in rice. Here, the results showed that three metabolites were upregulated in the phenylpropanoid biosynthesis pathway, namely, Com_684_pos (caffeic aldehyde), Com_1169_pos (scopoletin), and Com_621_pos (scopolin). This result showed that these three upregulated metabolites of the phenylpropanoid biosynthesis pathway might protect cucumber anther against high-temperature stress.

Plant hormones, such as auxin, gibberellin, abscisic acid, jasmonic acid, and salicylic acid, play a vital role in plant growth and development and abiotic stress tolerance. High temperature will affect hormone biosynthesis, the hormone signaling pathway, and hormone transport and causes a series of physiological changes (Castroverde and Dina, 2021). Auxin, which is a vital endogenous hormone, has an effect on plant growth and development under high-temperature stress (Luo et al., 2022). The content of auxin is higher under high-temperature stress, which affects male gametophyte development (Ding et al., 2017). Auxin is accumulated excessively under high-temperature stress, which damages hormone homeostasis and causes pollen abortion and male sterility (Khan et al., 2022b). According to our results, indole-3-acetic acid (Com_670_pos) was detected to be upregulated in high-temperature stress. In our previous study, a total of 16 differentially expressed genes were explored at the mature stage in high-temperature stress *vs*. normal condition, including auxin efflux carrier family protein, auxin-responsive protein, GH3 auxin-responsive promoter, and auxin-responsive SAUR protein (**Table S6**) (Chen et al., 2021b). The auxin efflux carriers (PIN5 and PIN8) are considered to regulate the balance and metabolism of auxin in pollen and also participate in pollen morphology development (Bosco et al., 2012; Ding et al., 2012). The abrupt expression of auxin-related genes caused changes in auxin content and led to a reduction in pollen fertility (Salinas-Grenet et al., 2018; Khan et al., 2022a). Moreover, jasmonic acid has an important role in biotic and abiotic stress responses. The altered expression of jasmonic acid biosynthesis-related genes, *GhACNAT* (*acyl-CoA N-acyltransferase*) from cotton, affects plant male fertility (Chen et al., 2021a). The exogenous application of jasmonic acid can alleviate male sterility in *GhACNAT*-silenced plants (Fu et al., 2015). The content of jasmonic acid in the anther changed and led to male sterility under high-temperature stress (Khan et al., 2020). In this study, jasmonic acid (Com_175_neg) was found upregulated in high-temperature stress. Two jasmonic acid-related genes (*CsaV3_5G039150* and *CsaV3_4G002460*) were detected to have a differential expression after high-temperature stress in our previous study (Chen et al., 2021b, **Table S6**). Furthermore, the deletion of the jasmonic acid biosynthesis gene allene oxide cyclase 2 (*GhAOC2*) in cotton led to H_2_O_2_ accumulation in the anthers and caused pollen abnormality and male sterility (Khan et al., 2022a). In addition, *CsaV3_5G039150*, an allene oxide cyclase protein, was upregulated after high-temperature stress. Jasmonate ZIM domain (JAZ) proteins can regulate the jasmonic acid signaling pathway and affect male fertility (Chung and Howe, 2009). In cucumber, *CsJAZ1* interacts with *CsGL2-LIKE* and plays a role in male flower development (Cai et al., 2020). *CsaV3_4G002460*, which encoded the JAZ protein, was downregulated after high-temperature stress. The upregulation of indole-3-acetic acid and jasmonic acid and the abrupt expression of related genes caused endogenous hormone imbalance and led to the abnormality of male flowers under high-temperature stress.

## Conclusions

The regulation mechanism of male cucumber flower development under high-temperature stress is unclear. In this study, lower pollen fertility, increased insufficient pollen grains, and delayed degradation of the tapetum were found in high-temperature stress. Many metabolites whose contents changed significantly were identified in the anther after high-temperature stress, such as auxin and jasmonic acid, amino acids and derivatives, and carbohydrates and derivatives. The differential metabolites involved in amino acid metabolism, sugar metabolism, and plant hormone signal transduction pathway maintain cell osmotic balance; however, failure of the starch synthesis and breaking down of endogenous hormone balance caused abnormality in the male flowers. These results provide valuable information and theoretical support for further studies on the mechanism of metabolic response to high-temperature stress in cucumber.

## Data availability statement

The original contributions presented in the study are included in the article/**Supplementary Material**. Further inquiries can be directed to the corresponding author.

## Author contributions

LC and YL designed the experiment. LC, ZL, WL, MW, JY, BJ and QP performed most of the experiments. LC wrote the paper. SX and SY edited the manuscript. All authors contributed to the article and approved the submitted version.
