# Supplementary material for: Responses of differential metabolites and pathways to high temperature in cucumber anther
Source: Front Plant Sci. 2023 Apr 14;14:1131735. doi: 10.3389/fpls.2023.1131735 (PMC10140443; doi:10.3389/fpls.2023.1131735)
Supplement: Supplementary file 1 [file DataSheet_1.docx]

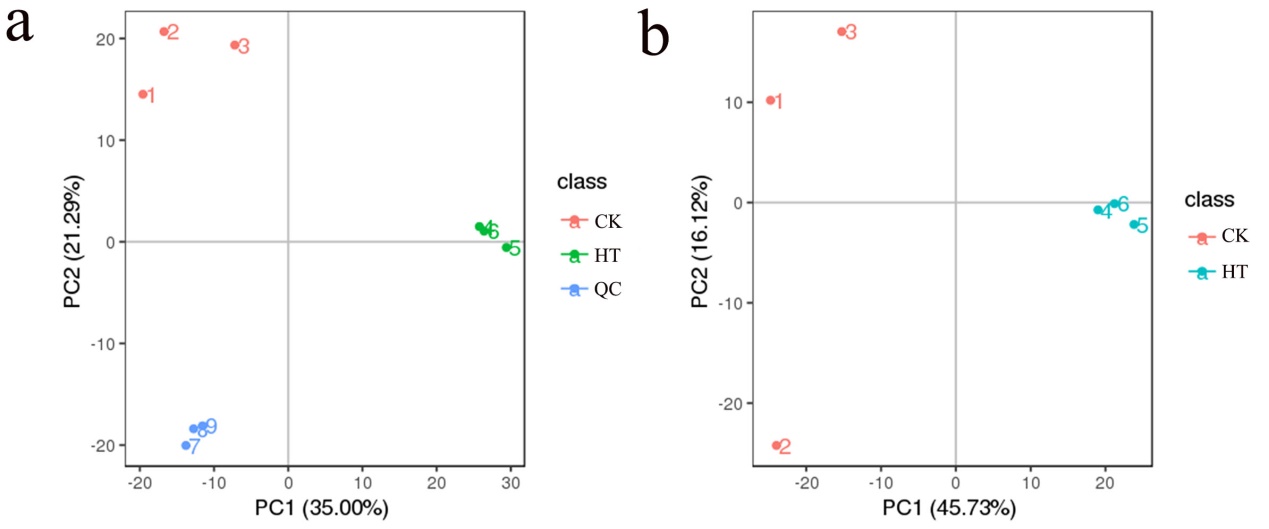


Figure S1. PCA of total samples (a) and PCA of CK and HT samples (b).


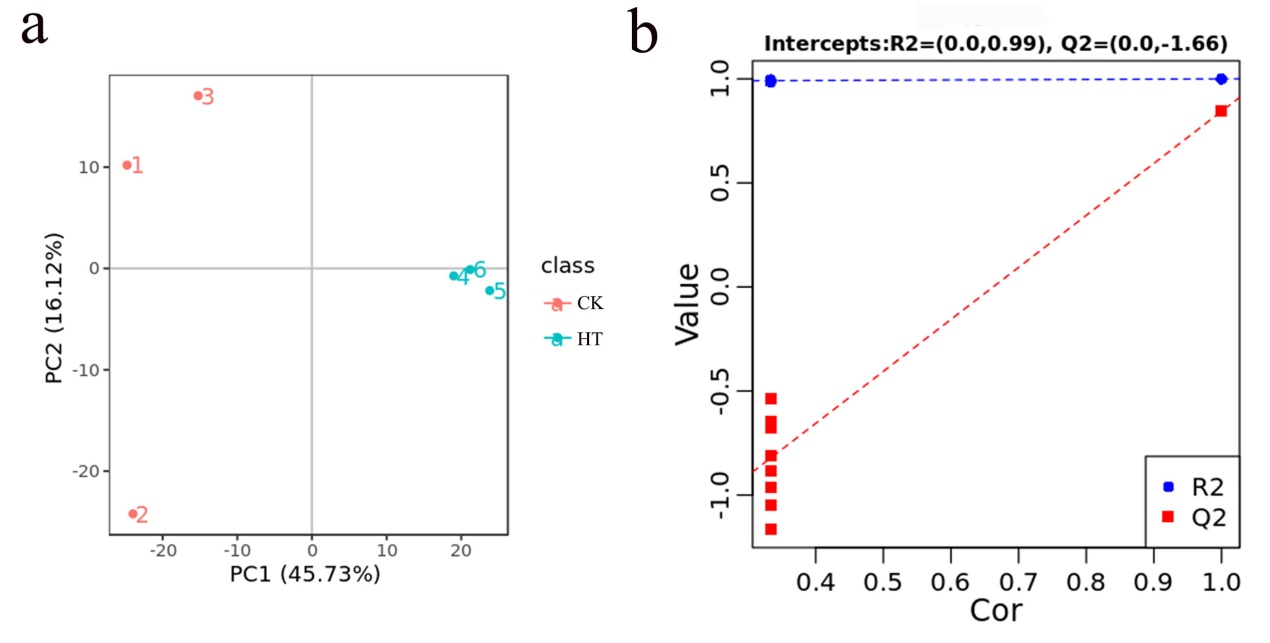


Figure S2 PLS-DA score plots and permutation test between high temperature stress and normal condition.

a, PLS-DA score plots; b, PLS-DA permutation test.
